# Clinical and Pathological Correlation in Pediatric Invasive Pulmonary Aspergillosis

**DOI:** 10.3389/fped.2018.00031

**Published:** 2018-02-21

**Authors:** Nattachai Anantasit, Noramon Nuntacharruksa, Pimpin Incharoen, Aroonwan Preutthipan

**Affiliations:** ^1^Division of Pediatric Pulmonology, Department of Pediatric, Faculty of Medicine, Ramathibodi Hospital, Mahidol University, Bangkok, Thailand; ^2^Division of Pediatric Critical Care, Department of Pediatric, Faculty of Medicine, Ramathibodi Hospital, Mahidol University, Bangkok, Thailand; ^3^Department of Pathology, Faculty of Medicine, Ramathibodi Hospital, Mahidol University, Bangkok, Thailand

**Keywords:** invasive pulmonary aspergillosis, pediatric, histopathology, definition, sensitivity

## Abstract

**Introduction:**

Invasive’ pulmonary aspergillosis (IPA) has been one of the major causes of mortality in immunocompromised patients. The gold standard method for a diagnosis of IPA is histopathological examination of the lung tissue; however, post-procedural bleeding limits the feasibility of lung biopsy. The European Organization for Research and Treatment of Cancer/Invasive Fungal Infections Cooperative Group and The National Institute of Allergy and Infectious Disease Mycoses Study Group (EORTC/MSG) defined IPA. The objective of this study was to validate the EORTC/MSG 2008 definition of IPA, compared with histopathology in the pediatric population.

**Methods:**

Histopathological examinations of lung tissues of children aged 1 month–18 years with respiratory tract infection at the time of obtaining biopsy were retrieved. Retrospective chart reviews for clinical characteristics were performed. IPA diagnosis was classified according to the EORTC/MSG 2008 definition.

**Results:**

During the 10-year period, there were 256 lung tissues, of which 58 specimens were suspected to have pulmonary infection. Fourteen patients (24%) were noted to have IPA. Seven patients (50%) with proven IPA were classified as probable, while the remaining 50% were classified as possible, and none were classified as no IPA, by using EORTC/MSG 2008 definition. Other 44 specimens demonstrated 14 (32%), 14 (32%), and 16 (36%) were classified as probable, possible, and no IPA, respectively. When comparing probable or possible IPA with no IPA, we found that the EORTC/MSG 2008 definition had 100% sensitivity, 36% specificity, 33% positive predictive value, and 100% negative predictive value in diagnosis of IPA.

**Conclusion:**

Our study illustrated that the EORTC/MSG 2008 definition provided an excellent sensitivity but low specificity for diagnosing IPA.

## Introduction

Invasive pulmonary aspergillosis (IPA) has been one of the major causes of mortality in immunocompromised patients, such as malignancy, hematopoietic stem cell transplantation, and prolonged usage of immunosuppressive agents. Early diagnosis and prompt treatment improve survival outcome (1, 2). Aspergillus species are ubiquitous in the environment. Aspergillus fumigatus is the most common species in IPA (3, 4). Other species include Aspergillus flavus, Aspergillus niger, and Aspergillus terreus. Aspergillus is introduced to the lower respiratory tract by inhalation of the infectious spores. In healthy hosts, spores are eliminated by mucociliary clearance and immune defense. In immunocompromised patients, dormant spores convert into growing hyphal elements and invade lung parenchyma and vascular structure (1). Clinical symptoms and signs of IPA are indistinguishable from other pathogens causing pneumonia. Chest radiography is also non-specific. Therefore, the diagnostic tool is challenging (5, 6).

The gold standard method in the diagnosis of IPA is histopathological examination of lung tissue obtained by bronchoscopy or open thoracotomy. The presence of angioinvasion by acute angle, branching septate fungal hyphae along with a positive culture for Aspergillus from the same site is diagnostic for IPA (1). However, invasive procedures hinder the possibility of obtaining lung tissues. The angioinvasive nature of Aspergillus and the patient condition such as thrombocytopenia further increase risk of bleeding and other complications.

In 2002, The European Organization for Research and Treatment of Cancer/Invasive Fungal Infections Cooperative Group and The National Institute of Allergy and Infectious Disease Mycoses Study Group (EORTC/MSG) formed a consensus committee to develop a standard definition for invasive fungal infections, including IPA. The definitions were intended for use in the context of clinical and/or epidemiological research, not for clinical decision making (7). These definitions were revised in 2008 due to the advances in diagnostic tools, including galactomannan, beta-D-glucan, and fungal DNA in body fluids by PCR (8, 9). Diagnosis of IPA is challenging, as the clinical symptoms are not specific. There is no single investigation for the diagnosis. IPA carries a high mortality rate if untreated, and delayed treatment can result in death. The ideal diagnostic tool should have high sensitivity for early recognition and treatment. In brief, based on EORTC/MSG 2008 consensus definition, there were three classifications; proven, probable, and possible. Proven IPA requires histopathology with a characteristic of infection and/or a positive culture of specimen from a sterile site. Probable IPA requires the fulfillment of criteria within three categories: host factors, clinical, and mycological criteria. Possible IPA consists of host factors, and clinical criteria without mycological evidence of Aspergillus infection (8). These definitions have been widely used in epidemiological research, and clinical trials for evaluation of new drugs and management strategies. However, they were based on the literature review, which did not include pathological results and was originally for the adult oncological population. To our knowledge, there were limited data about validity of the consensus definition compared with the histopathological diagnosis of IPA especially in the pediatric population. The objective of this study was to validate the EORTC/MSG 2008 consensus definition with the gold standard of histopathological results in pediatric patients.

## Materials and Methods

### Study Design and Purpose

**Figure 1:**
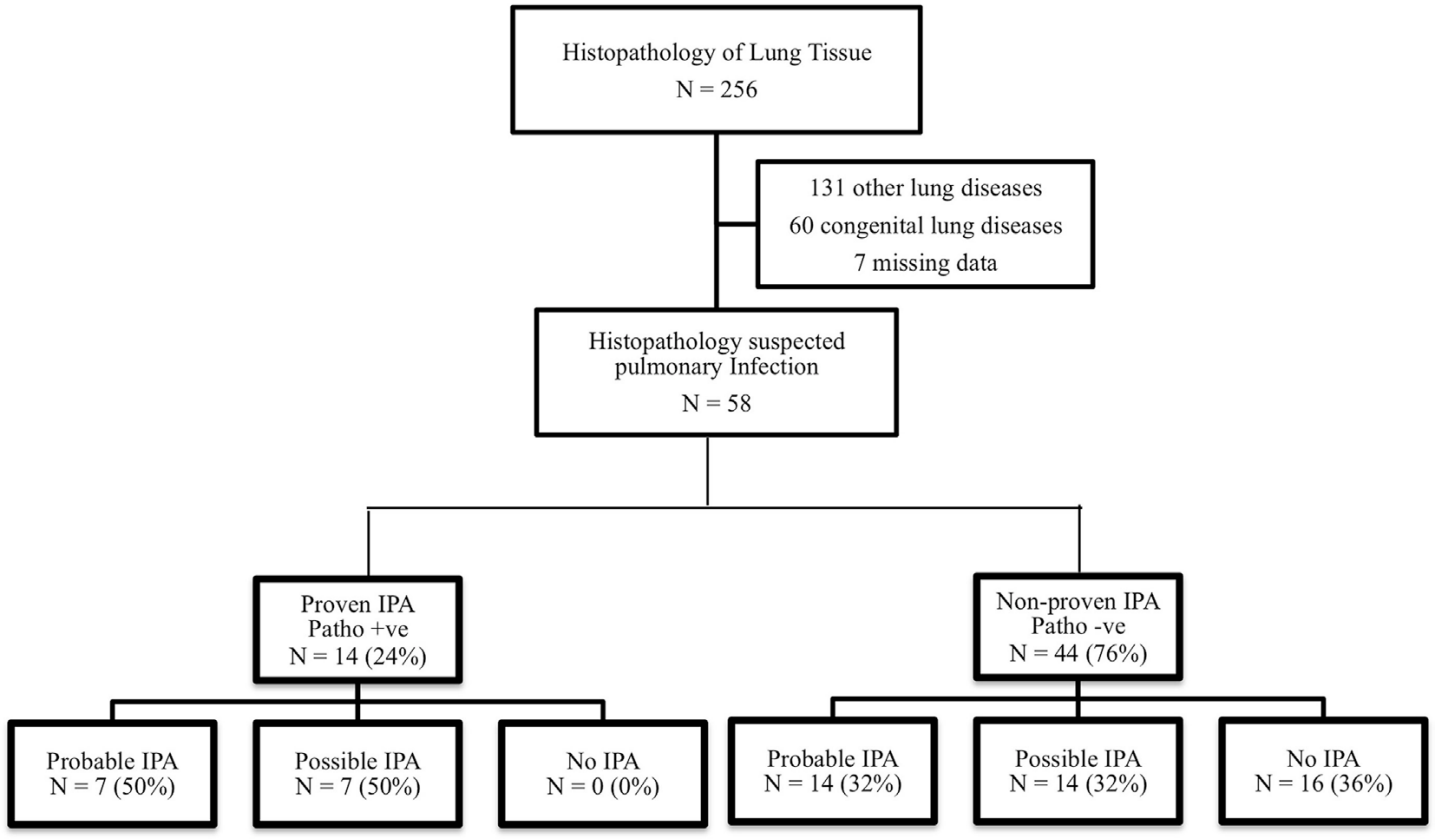
Flow diagram and classification of invasive pulmonary aspergillosis (IPA) according to the EORTC/MSG 2008 consensus definition.

This study was performed in a tertiary care, academic center. We retrospectively analyzed histopathology tissues in children aged 1 month–18 years with clinical suspicion of respiratory tract infection from January 2006 to December 2016. This study was approved by the Institutional Review Board. Patients with congenital lung disease, pulmonary malignancy, and incomplete medical records were excluded. Clinical characteristics, diagnostic tests, treatments, and pathological results were reviewed. Patients were categorized into two groups by the histopathological result as proven IPA and non-proven IPA and were classified according to the EORTC/MSG 2008 consensus definition into three groups (probable, possible, and no IPA) as shown in Figure 1.

### Definitions

The definition criteria for proven, probable, and possible IPA were shown in Table 1.

**Table 1:** The diagnostic criteria for IPA according to the EORTC/MSG 2008 consensus definition (8).

	Proven IPA	Probable IPA	Possible IPA
Positive for histopathological criteria – A discrete nodule consisting of necrotic lung tissue with angioinvasive by acute angle branching septate fungal hyphae or culture positive for Aspergillus spp. from lung tissue specimen	Yes	No	No
Positive for host factor criteria – Neutropenia – Post stem cell transplantation – Prolonged corticosteroid use – Received immunosuppressants – Inherited immunodeficiency		Yes	Yes
Positive for clinical criteria Presence of 1 of the following three signs on CT – Dense, well-circumscribed lesion with or without a halo sign – Air-crescent sign – Cavity		Yes	Yes
Positive for mycological criteria – Positive direct test by culture – Positive indirect tests by galactomannan antigen detection		Yes	No

*IPA; invasive pulmonary aspergillosis, CT; computer tomography*.

Proven IPA is characterized by a discrete nodule consisting of necrotic lung tissue with angioinvasion by acute angle, branching septate fungal hyphae or culture positive for Aspergillus spp. from lung tissue specimen (10, 11).

Non-proven IPA is defined as patients who do not meet the pathological criteria.

According to the EORTC/MSG 2008 consensus definition (8), clinical criteria in IPA consists of the presence of one of the following three signs on computer tomography; dense, well-circumscribed lesions with or without a halo sign, air-crescent sign, or cavity.

Positive clinical IPA is defined as patients who have clinical diagnosis with probable or possible IPA by the EORTC/MSG 2008 consensus definition.

Negative clinical IPA is defined as patients who do not meet criteria for diagnosis by the EORTC/MSG 2008 consensus definition.

Galactomannan enzyme immunoassay was measured from serum and bronchoalveolar lavage (BAL) fluid. Results were recorded as an index relative to the mean optical density of the threshold controls (GM index = optical density sample/mean optical density of the threshold control samples). Samples that had an index value >0.5 were considered positive for both serum and BAL fluid specimen (12, 13).

### Statistical Methods

SPSS software (version 18.0, SPSS Inc., Chicago, IL, USA) was used for statistical analysis. Patients’ demographics, clinical characteristics, and pathological data were analyzed by descriptive analysis using student’s t-test and Chi-Square. For the diagnostic test, sensitivity, specificity, positive predictive value, and negative predictive value were obtained. Correlation between clinical diagnosis and pathological result was calculated using Spearman rho correlation analysis. A p-value of less than 0.05 was considered statistically significant.

## Results

In total, 256 lung tissues were documented. Fifty-eight pathological specimens revealed suspicion of infectious etiologies, 14 (24%) patients were consistent with proven IPA, and 44 (76%) patients were non-proven IPA as shown in Figure 1. All patients who were diagnosed with probable or possible IPA by the EORTC/MSG 2008 definition (positive clinical IPA) received antifungal medications, although 28 out of 42 patients were not diagnosed with proven IPA, but received antifungal medications. Five of 16 patients who were not diagnosed with IPA by the EORTC/MSG 2008 definition (negative clinical IPA) received antifungal medications. None of these patients were diagnosed with proven IPA. The adverse events from antifungal medications were hypokalemia and acute kidney injury, although none of patients required renal replacement therapy. Baseline characteristics of histopathological proven IPA and non-proven IPA were showed in Table 2. There was no significant difference of demographic data in both groups. The proven IPA group had significantly more neutropenic patients than the non-proven IPA group (p-value = 0.009). The mean duration of neutropenia was longer in proven IPA than in non-proven IPA group. Mean was 22.1 ± 35.3 days in proven IPA and 12.9 ± 12.5 days in non-proven IPA group (p-value = 0.307).

**Table 2:** Baseline characteristics of the included patients.

	Proven IPA (*n* = 14)	Non-proven IPA (*n* = 44)	*p*-Value
Age, mean ± SD	10.4 ± 5.9	9.0 ± 6.1	0.438
Male (%)	4 (29)	21 (48)	0.207
Underlying disease, *n* (%)			0.064
– Malignancy	12 (86)	19 (43)	
– Primary immune deficiency	0 (0)	6 (14)	
– Connective tissue disease	0 (0)	3 (7)	
– Others	2 (14)	9 (20)	
– Healthy	0 (0)	7 (16)	
Neutropenia, *n* (%)	11 (79)	17 (39)	0.023*
Post transplantation, *n* (%)	1 (7)	6 (14)	0.108
Corticosteroid use, *n* (%)	1 (7)	4 (9)	0.904
Presentation symptom, *n* (%)			0.343
– Febrile neutropenia	9 (64)	18 (41)	
– Dyspnea	1 (7)	10 (23)	
– Hemoptysis	2 (14)	7 (16)	
– Others	2 (14)	9 (20)	
Serum galactomannan, median (IQR)	0 (0–2.2)	0 (0–1.4)	0.889
BAL galactomannan, median (IQR)	3.4 (1.5–7.5)	2.3 (0–4.0)	0.772
Computer tomography finding, *n* (%)			0.390
– Dense, well-circumscribed with/without Halo sign	11 (79)	26 (59)	
– Non-specific infiltration/consolidation	2 (14)	9 (21)	
– Air-crescent sign	1 (7)	1 (2)	
– Cavitation	0 (0)	7 (16)	
– Normal finding	0 (0)	1 (2)	
Exposure to beta-lactam antibiotics, *n* (%)	2 (14.3)	10 (22.7)	0.497
Antifungal treatment, *n* (%)			0.200
– Amphotericin B	6 (43)	22 (50)	
– Combination drugs	4 (29)	4 (9)	
– Voriconazole	3 (21)	6 (14)	
– Caspofungin	1 (7)	1 (2)	
– No antifungal	0 (0)	11 (25)	
Hospital mortality, *n* (%)	6 (43)	23 (52)	0.539

*IPA; invasive pulmonary aspergillosis, IQR; interquartile range*.

*p-Value less than 0.05.

### Microbiology and Pathological Results

Of the 58 lung tissue specimens, 18 lung tissues were obtained by open biopsy, 13 by necropsy, 10 by autopsy, 10 by CT/US guided needle biopsy, and 7 by transbronchial biopsy. The reasons for biopsy were for diagnosis in 28 (48%) patients, no response to antifungal medications or progression of diseases in 20 (35%) patients, and others in 10 (17%) patients. The mean duration from treatment to biopsy was 20.6 and 35.9 days in proven and non-proven IPA group, respectively (p-value = 0.431). The microbiology in non-proven IPA was virus in 7 patients (cytomegalovirus in 6 cases and respiratory syncytial virus in 1 case) and bacteria were found in 10 patients (3 cases were A. baumannii, 2 cases P. aeruginosa, 2 cases Actinomycoses spp.,1 case M. tuberculosis, and 2 cases other bacteria). Fungus was isolated in nine patients (four of Mucormycosis spp., two of Cryptococcosis spp., two of Pneumocystis spp., and 1 of Scedosporiosis spp.). The remaining 12 patients showed histopathologic change without identified pathogens, such as diffused alveolar damage and pulmonary hemorrhage, and 6 patients showed non-infectious histopathology.

### EORTC/MSG 2008 Consensus Definition

We analyzed clinical diagnosis of IPA by grouping probable and possible IPA as positive clinical IPA, and no IPA as negative clinical IPA. The sensitivity was 100% and the specificity was 36%. The positive and negative predictive values were 33 and 100%, respectively. Spearman’s rho correlation analysis revealed a correlation between clinical diagnosis and pathological result (r = 0.35, p-value = 0.008). Table 3 demonstrated each element in the EORTC/MSG 2008 consensus definition, in terms of sensitivity, specificity, positive, and negative predictive values.

**Table 3:** The sensitivity and specificity of each element in the EORTC/MSG 2008 consensus definition.

	Sensitivity (%)	Specificity (%)	PPV (%)	NPV (%)
– Host risk	100	15.9	27.5	100
– Computer tomography finding	85.7	22.7	26.1	83.3
– Culture	36.4	100	100	81.6
– Serum galactomannan	33.3	68.2	22.2	79
– BAL galactomannan	100	26.7	26.7	100

*PPV; positive predictive value, NPV; negative predictive value, BAL; bronchoalveolar lavage*.

## Discussion

This study validated the EORTC/MSG 2008 consensus definition with the gold standard histopathological tissue in pediatric population. Our results were consistent with previous studies revealing that proven IPA patients were found to be more neutropenic patients. The function of neutrophil is directed against Aspergillus hyphae (14). The neutropenia can increase risk of IPA. A prior study showed that cavitation or air-crescent sign in chest computed tomography is helpful in diagnosing IPA (15). Nevertheless, pediatric studies have demonstrated that nodules or infiltration are the most common radiologic findings in pediatric IPA (16, 17). Our study found no difference in radiologic findings in proven IPA and non-proven IPA groups. We had defined positive imaging as dense, well-circumscribed lesions with or without halo sign, air-crescent sign, or cavitation. The analysis showed high sensitivity but low specificity as in a previous study (18).

Galactomannan is a cell wall polysaccharide released by Aspergillus species during growth (19, 20). Serum galactomannan is more superior than BAL fluid because it is less invasive and provides a higher sensitivity (21). However, our study illustrated that the sensitivity of serum galactomannan was lower than BAL (33 vs. 100%, respectively) which was a similar result to another study (22). We hypothesized that since lungs are a primary site of IPA before angioinvasion may causes an elevation of BAL galactomannan prior to that of serum (23). In our study, the specificity of BAL fluid galactomannan was surprisingly low (27%) compared with previous studies (87.5–94%) using the same cutoff value (20, 24, 25). However, this study had included non-malignant patients which was different from the previous studies. In addition, this could be explained by using different classification as most studies used proven and probable as IPA instead of using histopathology as a gold standard. Another reason could be from the beta-lactam antibiotics. The beta-lactam antibiotics are derived from molds of the genus Penicillium. Galactomannan moieties are shared between Aspergillus and Penicillium species (26). Therefore, beta-lactam antibiotics have been reported to cause false positive of galactomannan.

To our knowledge, this study is the first study to validate the EORTC/MSG 2008 consensus definition in pediatric population with IPA by using histopathological tissues. Although the EORTC/MSG 2008 consensus definition was originally for the adult immunocompromised population. This study showed that the EORTC/MSG 2008 consensus definition had 100% sensitivity, but low specificity (36%), in diagnosing IPA when compared with histopathology in pediatric population. IPA has high mortality if left untreated, our paper showed the EORTC/MSG 2008 consensus definition can be helpful as the screening tool in pediatric populations. Therefore, children with probable or possible IPA according to this definition should be promptly treated with antifungal medications, monitored for adverse events of antifungal medications, and need confirmed diagnosis if there is no clinical response.

This study has some limitations. First, retrospective study might not provide all data needed especially serum and BAL galactomannan. Second, the prevalence of IPA might be underestimated because our study started with histopathological tissue, as we were unable to perform biopsy in every case of suspected IPA. In addition, some patients only underwent imaging guided biopsy or transbronchial biopsy, which might result in inadequate specimen. Therefore, it may not represent all pediatric IPA. The last limitation is that this was a single-center study with small sample size. A larger sample size of these groups would allow better results and analysis.

## Conclusion

Pediatric IPA is a life-threatening disease with high mortality. The diagnostic tool is an important method to early recognition and prompt treatment. Our study shows that the EORTC/MSG 2008 consensus definition provides an excellent sensitivity as screening tool, but low specificity for the diagnosis of IPA.

## Ethics Statement

This study was carried out in accordance with the recommendations of Committee on human rights related to research involving human subjects, Faculty of Medicine Ramathibodi Hospital, Mahidol University.

## Author Contributions

NN and NA contributed to design of the study, data collection, data analysis, and manuscript drafting. PI and AP contributed to design of the study. NA critically revised it for important intellectual content. All authors gave final approval of the version to be published.

## Conflict of Interest Statement

All authors: there are no potential conflicts of interest. All authors have submitted the ICMJE Form for Disclosure of Potential Conflicts of Interest. Conflicts that the editors consider relevant to the content of the manuscript have been disclosed.
